# Design and validation of a food frequency questionnaire (FFQ) for the nutritional evaluation of food intake in the Peruvian Amazon

**DOI:** 10.1186/s41043-019-0199-8

**Published:** 2019-12-19

**Authors:** María García Rodríguez, Manuel Romero Saldaña, José Manuel Alcaide Leyva, Rafael Moreno Rojas, Guillermo Molina Recio

**Affiliations:** University of Córdoba, Córdoba, Spain

**Keywords:** Food frequency questionnaire, Dietary intake, Peruvian Amazon, Nutritional epidemiology

## Abstract

**Background:**

Food frequency questionnaires (FFQs) are dietary surveys widely used in studies of nutritional epidemiology because they are inexpensive, easy to complete and provide significant information on dietary intake over a long period of time. FFQs should be written specifically for each study group since diet may be influenced by ethnicity, culture, economic status and environmental factors. The aim of the authors on this work was to design and validate a food frequency questionnaire for the Peruvian Amazon population.

**Methods:**

Three hundred three individuals were selected and completed three 24-h recalls (R24). Two FFQs were conducted on two different occasions (FFQ.1 and FFQ.2). The validity of the FFQ was evaluated by comparing the relationship between the average daily nutrient intake estimated by the FFQs and that obtained by the three R24. The reproducibility was measured by comparing the mean nutrient intake of the two FFQs carried out. The correlations were assessed using the Pearson correlation coefficient, the intraclass correlation coefficient (ICC), the Lin correlation coefficient (CCC) and the Bland–Altman plot.

**Results:**

The results obtained to establish the validity showed a high correlation, with an average Pearson’s correlation coefficient of 0.70, a CCI of 0.65 and a CCC of 0.60. Approximately 60% of the nutrients had a CCC above 0.60. In terms of reproducibility, better results were obtained, with an average Pearson’s correlation coefficient of 0.67, 0.64 for CCI and 0.58 for CCC.

**Conclusions:**

The correlation coefficients show good validity and reproducibility, and therefore, the FFQ we have developed may be considered a useful and valid tool to estimate the dietary intake of the Peruvian Amazon population.

## Background

Dietary intake is one of the determining factors in the development of chronic diseases. Unlike other habits, it is a complex value to measure because people are rarely able to discriminate exactly what and how much they eat [1, 2]. In addition, an inaccurate dietary assessment can often be a major obstacle to understand the impact on the risk of developing diet-related diseases [2].

A range of different tools, from 24-h recalls (R24) or dietary diaries to food frequency questionnaires (FFQ), are used nowadays to measure dietary intakes. FFQs are the most commonly employed tools in studies of nutritional epidemiology because they are inexpensive, easy to develop and provide useful information on dietary intake over a long period of time [3–5]. Furthermore, this tool can be used to conduct personal interviews. This is extremely important when studying population groups with high illiteracy rates.

The FFQ should be developed specifically for the target population of the study since diet may be influenced by ethnicity, culture, economic status and environmental factors [6]. Besides, the questionnaire must be validated to ensure that the measurements are correct and therefore provide relevant information [7]. To assess the validity of the measurements obtained, it is necessary to check that the results obtained are similar to the ones observed when employing other methods [8–10].

The Department of Loreto is the largest territory in the Peruvian Amazon. Its capital city is Iquitos, located in the Great Plains of the Amazon Basin on the banks of the Amazon River at the confluence of the Nanay and Itaya Rivers. According to the data from the Peruvian National Institute of Statistics and Informatics (INEI) in 2015 [11], “this province has been placed in the third group in the range of extreme poverty incidence, with a high rate of chronic malnutrition in children and of overweight and persistent diseases in adults”. According to diverse studies, the population of Loreto has not been eating properly for a considerable period of time [12–15]. Given the amount of food resources available in this area [14], this situation can be put down to a poor food culture along with a low purchasing power [15]. For this reason, conducting dietary intake studies represent a fundamental step to know the vulnerability of the population of Loreto and the severity of the problems associated with their nutrition. At the same time, not many researches have focused on assessing intake and eating patterns in this region. It is important to highlight that its geographical location, the climate, the large number of existing indigenous people and its border situation with three other countries (Ecuador, Colombia and Brazil) provide significant differences in the eating habits of its inhabitants with respect to other regions of Peru [14, 15].

However, in Peru, the FFQ currently in use is not suitable for this area since it is made up of foods commonly consumed in urban areas regardless of food availability, consumption and eating habits of the people who live in the jungle.

The objective of this work is, therefore, to design and validate a new FFQ specifically aimed to study food intake habits of the Peruvian Amazon population analysing their food consumption patterns in greater depth and identifying possible deficiencies and their relationship with chronic diseases.

## Methods

### Study design, population and sample

We carried out an observational survey study divided in three phases. The target population was made up of the inhabitants of Pueblo Libre, a populated centre of Belén located in the periphery of Iquitos, an area affected by floods between February and June. This location lends it a transitional character between the communities who live close to the rivers in the jungle and the city itself. It was selected for this study because it clearly represents this transition and because it allows us to obtain more information about the local foods consumed in the area.

The statistical-epidemiological package EPIDAT (version 4.1) and the Pueblo Libre population census (Belén District, Iquitos, 2009) were used to determine the sample size [16]. For an expected prevalence of chronic malnutrition of 24.9% [17], an accuracy level of 5% and a safety level of 95%, a minimum sample size of 218 persons was obtained.

Stratified random sampling without replacement was performed by age and gender based on the random selection of 40 homes corresponding to Sector 12 of Pueblo Libre. It was assumed that the family unit of each home was made up of an average of 6 people. In the end, a total of 303 individuals were selected aged between 3 and 83 years old.

### Reference method

To design the FFQ and its subsequent validation, the R24 was taken as the reference method. This type of dietary survey provides information not only about an individual’s food consumption on a given day but also about the amounts of food ingested, the ingredients and the cooking method used on each recipe. Besides, since it is carried out as an interview, it is an ideal method for population groups with a low literacy level, like in this case [18].

For each studied individual in the sample, three R24 were collected over three consecutive days: two on business days and one on a non-working day. The R24 were conducted through a personal interview by a qualified and trained nutritionist at each participant’s home. In the case of children, their mothers were interviewed. The interviewees explained the amounts of food they had consumed using domestic measurement units (a tablespoon, a cup, etc.). These were shown to the interviewer, who then estimated the weight in grams of every portion. The R24 were given to the same person on all occasions to avoid variability in food intake.

After that, the average amounts of food, energy and nutrients ingested were calculated using Nutriplato 4.7 software [19]. It was updated with information from the Peruvian Amazon food composition database compiled by us and the nutritional details of the dishes most frequently consumed by this population group [15].

### Food frequency questionnaire

To design and validate our FFQ, we developed a food composition database specifically for the Peruvian Amazon population. We used it together with the FFQ that was being used in other areas of Peru [15]. We omitted non-typical Amazon foods and added other commonly consumed ones in the studied area. It was composed of 132 food types classified in 10 categories ((1) dairy products; (2) eggs, meat, fish and seafood; (3) vegetables; (4) fruits; (5) cereals, legumes and dry fruits; (6) fats and oils; (7) creams; (8) sweets and snacks; (9) drinks; and (10) condiments). The amount of food consumed was calculated according to the amounts consumed on a daily, weekly, or monthly basis. The weight in grams of the average servings was estimated using the mean of consumption obtained in the R24 (Additional file 1).

**Fig. 1:**
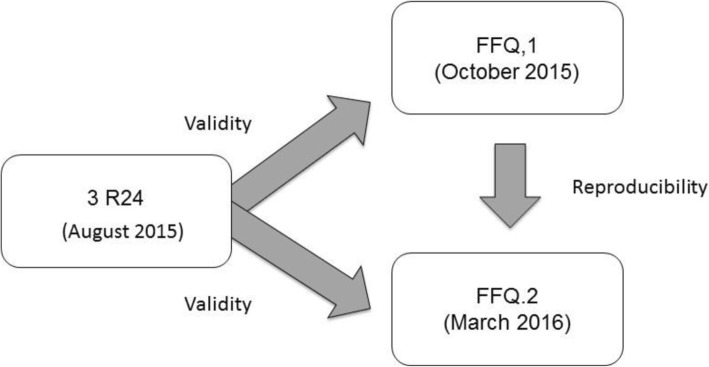
Validation process of FFQ for the Peruvian Amazon population

One hundred six of the 303 individuals who completed the R24 went through the FFQ personal interview twice: firstly, 2 months after the R24 completion and a second time, 5 months later (Fig. 1).

The average food, energy and nutrient consumption estimate was also performed using the updated Nutriplato 4.7 [19] software.

The FFQs were given to the same person on all occasions to avoid variability in food intake.

### Validation and analysis of the data

The quantitative variables were analysed according to their mean, median and standard deviation (SD) for the R24 and FFQ. The importance of the differences in nutrient intake between the FFQ assessments and the mean value of the three R24 was carried out using the Wilcoxon test or *t* test for related samples, according to the distribution of the data. Gross classification errors were calculated using contingency tables for the average intake value of the three R24 and the FFQ.1 since this one was the closest in time.

To evaluate the validity of the FFQ, we compared the correlation between the average daily nutrient intake values obtained for the two FFQs and that obtained for the three R24 [4, 20–22]. Histograms with normality curves and the Kolmogorov-Smirnov test were used, considering *p* > 0.05 to verify the normality of the distribution of each nutrient. The data (log_10_) of the variables which did not comply were transformed. Pearson’s correlation coefficient was used to obtain an initial approximation of the correlation between the variables. Next, the intraclass correlation coefficient (ICC) was calculated [23, 24] in order to correct and eliminate inter- and intra-individual variations. To do this, the data were adjusted for energy according to the residual method proposed by Willet [24, 25] to control the confounding effect of the calories. Finally, Lin’s concordance correlation coefficient (CCC) was used to compute both the accuracy of the FFQ and the accuracy of the relation. This enabled us to observe the deviation of the data obtained by the two methods employed on a line from the origin and at 45°, corresponding to the perfect line of concordance or perfect reproducibility.

The Bland–Altman plot was used for energy and adjusted macronutrients [26, 27] to check graphically the limits of agreement between the measurements carried out by the R24 and FFQ.

The reproducibility assessment of the FFQ was performed by measuring the correlation between the two FFQs performed, with a 5-week difference, as well as using the Pearson correlation coefficient, the CCI and the CCC [8, 23, 25].

The calculations were made using the SPSS programme, version 15.0 (SPSS Inc., Chicago, USA). In all statistical tests, the significance level used was *p* < 0.05.

## Results

### Characterisation of the sample

Three hundred three individuals were selected between 3 and 83 years old, thus exceeding the limit of the previously calculated sample size which was 218.

Regarding gender distribution, the balance was almost exact: 156 men (51.3%) and 147 women. These data coincide with the gender structure used in 2009 [16] (Table 1).

**Table 1:** Gender distribution of the sample

	Frequency	Percentage
Men	156	51.3
Women	147	48.7
Total	303	100.0

As for the age and gender distribution, the population structure has also been respected. If we group this variable in the same age strata, we find that it follows a distribution similar to the one published in the census (Table 2).

**Table 2:** Distribution of the sample by age and gender groups

Gender	Frequency	Percentage
Men		
3–5 years old	22	13.9
6–9 years old	17	11.1
Men 10–13 years old	4	2.8
Men 14–19 years old	39	25.0
Men 20–29 years old	17	11.1
Men 30–39 years old	26	16.7
Men 40–49 years old	23	13.9
Men 50–59 years old	4	2.8
Men 60–69 years old	4	2.8
Total	156	100.0
Women		
3–5 years old	10	6.8
6–9 years old	20	13.6
Women 10–13 years old	33	22.7
Women 14–19 years old	17	11.4
Women 20–29 years old	28	18.2
Women 30–39 years old	17	11.4
Women 40–49 years old	14	9.1
Women 50–59 years old	8	4.5
Women 60–69 years old	3	2.3
Total	147	100.0

The mean, median and SD of nutrient intakes are shown in Table 3. The values estimated by the FFQs were significantly higher than those of the R24 for calcium, phosphorus, potassium, iron, folic acid and vitamins B12, C and D. No significant differences were found for macronutrients and energy. Regarding the comparison of intake between both FFQs, no significant differences were found, except for calcium, phosphorus and riboflavin.

**Table 3:** Daily intake of nutrients estimated by the average 3 R24, FFQ.1 and FFQ.2

Energy and nutrients	R24			FFQ.1			FFQ.2		
	Mean	Median	SD	Mean	Median	SD	Mean	Median	SD
Energy (Kcal)	1988.2	1924.5	751.4	2058.1	1990.5	846.8	2069.8	2006.5	809.8
Proteins (g)	96.6^b^	82.2	45.9	105.2	89.8	45.7	107.8	100.3	50.7
Fats (g)	88.1^a^	91.2	28.7	88.8	92.1	30.6	90.2	91.9	32.3
Carbohydrates (g)	273.3^b^	244.5	122.6	302.4	287.2	130.1	308.1	286.5	146.0
Ca (mg)	535.4	518.7^ªb^	221.4	634.9	551.9^b^	345.3	924.4	886.3	367.8
P (mg)	1028.3	879 ^ªb^	506.8	1274.8	1140.5^b^	651.2	1362.8	1143	697.2
Na (mg)	3909.0	3133.5	2067	4513.3	3809.5	2346	4414.4	3672.5	2206
K (mg)	2482.3	2720^ªb^	1284.6	2779.6	2376.5	1343.7	2840.9	2720	1284.6
Fe (mg)	7.8	7.5^ªb^	2.7	9.0	8.5	4.1	9.0	8.9	3.9
Tiamin (mg)	1.7	1.7^a^	0.9	1.9	1.8	0.9	1.9	1.8	0.8
Riboflavin (mg)	2.1	1.7^ab^	1.5	2.3	2.0^b^	2.5	1.2	0.7	1.4
Vitamin B6 (mg)	1.9	1.7^a^	1.1	2.0	2.0	1.1	2.0	1.7	1.2
Folic Acid (μg)	198.4	155.1^ab^	134.5	227.9	216.1	126.8	217.0	165.9	134.1
Vitamin B12 (μg)	4.4	2.5^ab^	4.0	6.9	6.0^b^	3.7	5.4	4.0	4.4
Vitamin C (mg)	116.7	92.8^ab^	72.4	146.7	129.0	84.0	134.1	123.2	73.5
Vitamin A (μg of RE)	320.4	314.0^a^	124.1	594.6	346.0	878.3	572.3	305.2	1056.5
Vitamin D (μg)	1.0	0.5^ab^	1.8	1.0	0.7	0.7	1.0	0.8	0.7

^a^Significant differences (*p* < 0.05) for FFQ.1

^b^Significant differences (*p* < 0.05) for FFQ.2

^ab^Significant differences (p < 0.05) for both FFQ

The percentages of classification errors are shown in Table 4. In both FFQs and the R24, between 60% and 96% of the individuals were classified in the same quintile or the adjacent one.

**Table 4:** Percentages of energy and nutrient classification errors

Item	Lower quintile in R24 and higher in FFQ (%)	Higher quintile in R24 and lower in FFQ (%)	Classified in same quintile or adjacent in FFQ and R24 (%)
Energy	0	0	95.4
Proteins	0	0	95.5
Fats	0	0	94.3
Carbohydrates	0	0	78.3
Calcium	0.9	0.9	77.5
Phosphorum	0	0.9	74.5
Sodium	0	0	94.3
Potasium	0	0	82.9
Iron	0.9	0	74.6
Tiamin	0	0	91.5
Riboflavin	2.8	4.7	64.3
Vitamin B6	0	0	86.9
Folic Acid	0	0	88.6
Vitamin B12	0	0.9	83.0
Vitamin C	0	0	84.1
Vitamin A	1.9	3.8	60.4
Vitamin D	4.7	2.8	50.1

### Validity

The validity results for energy and nutrient intake between R24 and FFQ.1 are shown in Table 5. After analysing Pearson’s correlation coefficient, ranges were found from 0.65 to 0.87 for macronutrients, 0.55–0.89 for minerals and 0.12–0.83 for vitamins. The highest correlations were obtained for energy, sodium, thiamine, vitamin B6 and folic acid and the lowest correlations were found for vitamins A and D. The ICC ranged from − 0.25 to 0.88 with values above 0.60 for all nutrients except for calcium, iron and vitamins A and D, with unadjusted data. When adjusted for energy, the ICC values were lower for many nutrients, although nearly all showed a correlation greater than 0.6. The values for Lin’s concordance correlation coefficient were higher for macronutrients and energy, with values between 0.76 and 0.89, 0.44 and 0.81 for minerals and 0.09 and 0.76 for vitamins. In all cases, the values obtained for vitamins A and D showed the lowest correlation.

**Table 5:** Nutrient intake validity between R24 and FFQ.1

Energy and nutrients	*r*^a^	ICC^b^ (CI 95%)		CCC^c^ (CI 95%)
		Unadjusted	Adjusted	
Energy (Kcal)	0.874**	0.866 (0.809–0.907)		0.865 (0.810–0.905)
Proteins (g)	0.649**	0.619 (0.478–0.854)	0.892 (0.846–0.926)	0.892 (0.845–0.925)
Fats (g)	0.772**	0.771 (0.681–0.839)	0.875 (0.822–0.913)	0.874 (0.821–0.913)
Carbohydrates (g)	0.815**	0.794 (0.682–0.864)	0.763 (0.643–0.841)	0.761 (0.671–0.967)
Ca (mg)	0.654**	0.564 (0.392–0.692)	0.510 (0.333–0.648)	0.508 (0.384–0.613)
P (mg)	0.713**	0.654 (0.415–0.788)	0.499 (0.156–0.698)	0.496 (0.370–0.604)
Na (mg)	0.889**	0.825 (0.680–0.897)	0.810 (0.660–0.887)	0.809 (0.736–0.863)
K (mg)	0.804**	0.855 (0.747–0.912)	0.804 (0.678–0.876)	0.802 (0.725–0.859)
Fe (mg)	0.548**	0.478 (0.307–0.618)	0.443 (0.277–0.584)	0.441 (0.307–0.558)
Tiamin (mg)	0.879**	0.861 (0.675–0.914)	0.233(− 0.034–0.593)	0.231 (0.174–0.288)
Riboflavin (mg)	0.796**	0.880 (0.807–0.923)	0.408 (0.203–0.572)	0.406 (0.260–0.534)
Vitamin B6 (mg)	0.820**	0.807 (0.718–0.868)	0.764 (0.664–0.835)	0.762 (0.671–0.831)
Folic Acid (μg)	0.829**	0.806 (0.698–0.873)	0.760 (0.639–0.840)	0.758 (0.668–0.827)
Vitamin B12 (μg)	0.578**	0.528 (0.164–0.727)	0.774 (0.681–0.842)	0.772 (0.682–0.839)
Vitamin C (μg)	0.769**	0.745 (0.504–0.857)	0.708 (0.452–0.832)	0.706 (0.608–0.783)
Vitamin A (μg)	0.120	0.025(−0.148–0.201)	0.08(− 0.163–0.185)	0.09(− 0.028–0.045)
Vitamin D (μg)	0.141	− 0.025(− 0.217–0.167)	0.08(− 0.163–0.144)	0.096(− 0.082–0.267)

^a^*r*, Pearson’s correlation coefficient, level of statistical significance: *P < 0.05, **P < 0.01

^b^ICC intraclass correlation coefficient

^c^*CCC* Lin’s concordance correlation coefficient

Table 6 shows the validity results between R24 and FFQ.2. The Pearson correlation coefficient produced a value of 0.87 for energy. For macronutrients, a range from 0.77 to 0.88 was observed, with minerals between 0.21 and 0.91 and vitamins between − 0.11 and 0.84. The ICC for unadjusted data was 0.86 for energy, between 0.77 and 0.84 for macronutrients, from 0.10 to 0.87 for minerals (with calcium showing the lowest correlation) and between − 0.026 and 0.85 for vitamins. Except for calcium, phosphorus, iron and vitamins A and D, all the nutrients obtained an ICC above 0.6. After adjusting the data, minimal variations were observed and the results were the same as for the unadjusted data, being calcium, iron and vitamins A and D the micronutrients that obtained the lowest values. The values for CCC were higher for macronutrients and energy: between 0.84 and 0.88. For minerals, very different data were obtained: 0.04 for calcium, 0.38 for iron, 0.72 for potassium and 0.86 for sodium. In the case of vitamins, only vitamin C, folic acid and vitamin B12 obtained a CCC higher than 0.6. Vitamins A and D were the ones that obtained the lowest values: 0.02 and − 0.07, respectively.

**Table 6:** Nutrient intake validity between R24 and FFQ.2

Energy and nutrients	*r*^a^	ICC^b^ (CI 95%)		CCC^c^ (CI 95%)
		Unadjusted	Adjusted	
Energy (Kcal)	0.871**	0.864 (0.806–0.906)		0.863 (0.807–0.904)
Proteins (g)	0.772**	0.781 (0.668–0.854)	0.884 (0.827–0.922)	0.883 (0.834–0.919)
Fats (g)	0.775**	0.769 (0.679–0.837)	0.840 (0.773–0.888)	0.838 (0.773–0.886)
Carbohydrates (g)	0.880**	0.840 (0.706–0.906)	0.855 (0.766–0.907)	0.853 (0.794–0.897)
Ca (mg)	0.212*	0.102(− 0.056–0.268)	0.046(− 0.058–0.167)	0.045(− 0.038–0.128)
P (mg)	0.731**	0.589 (0.259–0.762)	0.050(− 0.054–0.172)	0.049(− 0.028–0.126)
Na (mg)	0.911**	0.872 (0.752–0.927)	0.865 (0.741–0.923)	0.864 (0.809–0.904)
K (mg)	0.773**	0.773 (0.629–0.857)	0.723 (0.553–0.824)	0.721 (0.622—0.798)
Fe (mg)	0.504**	0.450 (0.277–0.593)	0.383 (0.195–0.542)	0.381 (0.242–0.505)
Tiamin (mg)	0.808**	0.798 (0.710–0.860)	0.207(− 0.046–0.548)	0.205 (0.148–0.261)
Riboflavin (mg)	0.907**	0.834 (0.738–0.958)	0.378 (0.04–0.743)	0.376 (0.300–0.446)
Vitamin B6 (mg)	0.764**	0.818 (0.742–0.873)	0.786 (0.701–0.849)	0.784 (0.701–0.847)
Folic Acid (μg)	0.843**	0.858 (0.792–0.895)	0.846 (0.772–0.895)	0.844 (0.781–0.891)
Vitamin B12 (μg)	0.628**	0.747 (0.629–0.827)	0.824 (0.752–0.877)	0.823 (0.751–0.876)
Vitamin C (μg)	0.751**	0.765 (0.647–0.843)	0.700 (0.570–0.792)	0.698 (0.589–0.782)
Vitamin A (μg)	− 0.11	− 0.11(− 0.190–0.173)	0.019(− 0.199–0.165)	− 0.019(− 0.05–0.012)
Vitamin D (μg)	0.08	− 0.026(− 0.218–0.166)	− 0.069(− 0.218–0.094)	− 0.068(− 0.154–0.019)

^a^*r*, Pearson’s correlation coefficient, level of statistical significance: *P < 0.05, **p < 0.01

^b^*ICC* Intraclass correlation coefficient

^c^*CCC* Lin’s concordance correlation coefficient

**Fig. 2:**
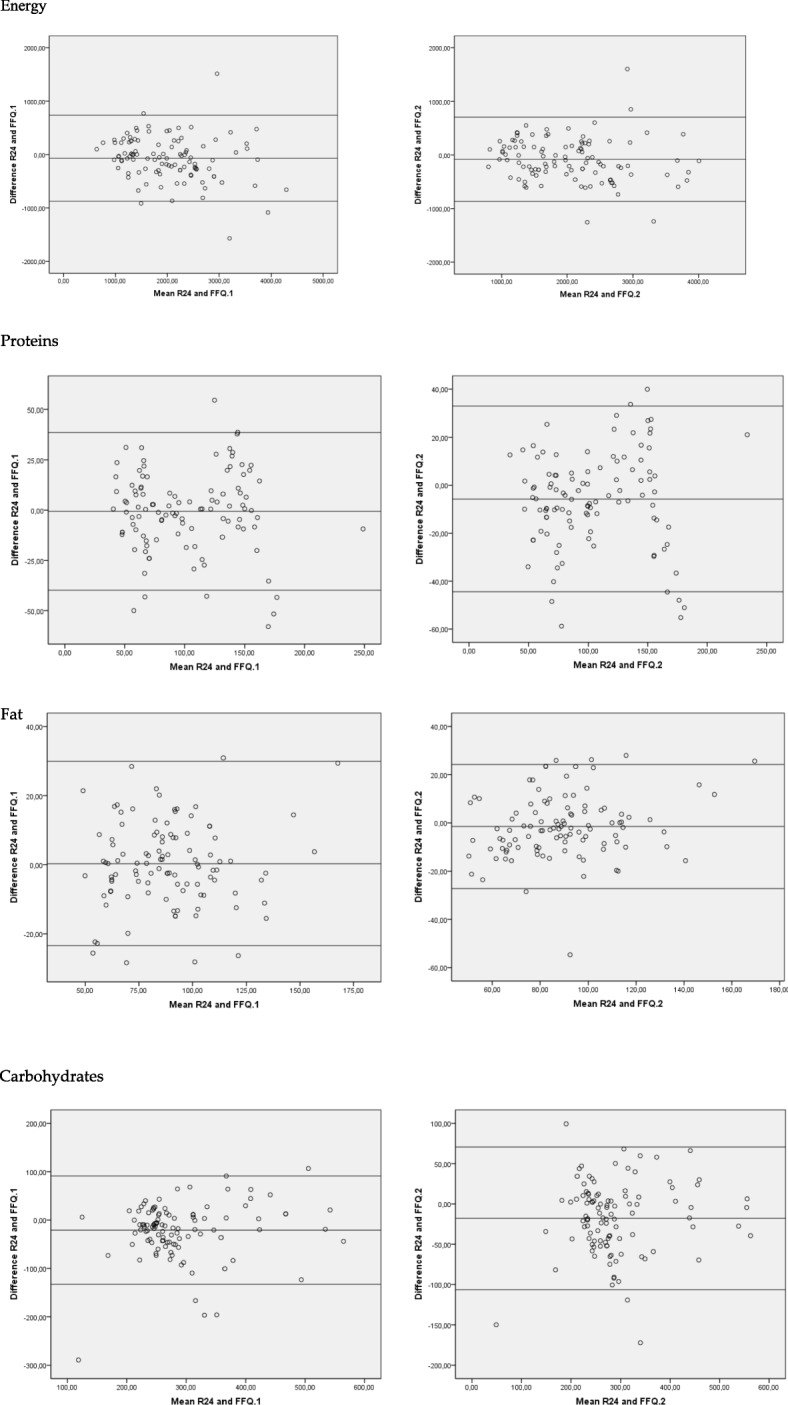
Bland–Altman plots. Validity

The Bland–Altman plots indicated a high level of agreement between both methods (Fig. 2) since there were very few observations outside the limits for energy, protein, lipids and carbohydrates.

### Reproducibility of the FFQ

The Pearson correlation coefficient showed very high values for energy and macronutrients (0.61–0.87). In the case of minerals, ranges between 0.22 and 0.95 were obtained, with calcium at the bottom end and those of sodium and phosphorus at the top. The ICC gave a value of 0.87 for energy, a range from 0.61 to 084 for unadjusted macronutrients and from 0.80 to 0.91 for adjusted macronutrients. In the case of minerals, the values ranged between 0.12 and 0.93 for non-adjusted data and between 0.12 and 0.91 for adjusted data, with calcium obtaining the lowest results in both cases. CCC showed very high values most of which were above 0.6, being energy, proteins, lipids, sodium and potassium, the ones showing the greatest concordance. Again, calcium and vitamins A and D obtained the lowest values (Table 7).

**Table 7:** Nutrient intake reproducibility between FFQ.1 and FFQ.2

Energy and nutrients	*r*^a^	ICC^b^ (CI 95%)		CCC^c^ (CI 95%)
		Unadjusted	Adjusted	
Energy (Kcal)	0.877**	0.877 (0.825–0.915)		0.876 (0.824–0.914)
Proteins (g)	0.609**	0.608 (0.473–0.715)	0.906 (0.858–0.937)	0.905 (0.864–0.934)
Fats (g)	0.751**	0.751 (0.655–0.824)	0.850 (0.787–0.895)	0.849 (0.787–0.894)
Carbohydrates (g)	0.843**	0.838 (0.771–0.887)	0.799 (0.718–0.859)	0797 (0.716–0.857)
Ca (mg)	0.224*	0.128(− 0.001–0.382)	0.120(− 0.043–0.285)	0.119(− 0.016–0.250)
P (mg)	0.950**	0.767 (0.675–0.836)	0.238 (0.036–0.419)	0.236 (0.106–0.359)
Na (mg)	0.947**	0.926 (0.894–0.949)	0.915 (0.877–0.941)	0.914 (0.877–0.940)
K (mg)	0.753**	0.787 (0.703–0.850)	0.710 (0.601–0.793)	0.708 (0.599–0.791)
Fe (mg)	0.717**	0.717(0.610–0.798)	0.691(0.577–0.779)	0.689(0.575–0.776)
Tiamin (mg)	0.760**	0.759(0.665–0.829)	0.667(0.547–0.761)	0.665 (0.545–0.758)
Riboflavin (mg)	0.683**	0.672(− 0.060–0.883)	0.230(− 0.085–0.500)	0.228 (0.133–0.319)
Vitamin B6 (mg)	0.778**	0.791 (0.707–0.853)	0.718 (0.605–0.801)	0.716 (0.612–0.794)
Folic Acid (μg)	0.783**	0.756 (0.661–0.827)	0.711 (0.603–0.794)	0.709 (0.600–0.792)
Vitamin B12 (μg)	0.628**	0.582 (0.401–0.711)	0.747 (0.631–0.828)	0.746 (0.650–0.972)
Vitamin C (μg)	0.742**	0.728 (0.622–0.807)	0.658 (0.532–0.755)	0.656 (0.539–0.784)
Vitamin A (μg)	0.121	0.005(− 0.187–0.196)	0.012(− 0.181–0.203)	0.09(− 0.028–0.045)
Vitamin D (μg)	0.188	0.190(− 0.001–0.368)	0.096(− 0.082–0.273)	0.002(− 0.009–0.013

^a^*r*, Pearson’s correlation coefficient, level of statistical significance: *P < 0.05, **P < 0.01

^b^*ICC* Intraclass correlation coefficient

^c^*CCC* Lin’s concordance correlation coefficient

The Bland–Altman plots showed a high level of agreement between both FFQs, since very few observations were found outside the limits.

## Discussion

An FFQ was designed and validated to make a nutritional assessment of food intake in the Peruvian Amazon. To determine the validity, the average nutrient intake was obtained using and comparing three R24 on consecutive days. As found in many other studies [3, 9, 20, 25, 28–32], R24 was chosen as the reference method. To determine reproducibility, the FFQ data were compared in two different occasions, with a time interval of 5 months between both moments. Several authors have indicated that to ensure minimum variation between the results obtained from two surveys collected from the same individual, there must be an intervening time period from 4 to 6 months [2, 26].

Regarding the distribution of the sample, it should be noted that there are not many individuals aged over 50 included in the age groups due to the low life expectancy of the population in this area as shown in the last census [11, 12, 16].

The results obtained to establish the validity show a close correlation, with an average Pearson’s correlation coefficient of 0.70, an average ICC of 0.65 and 0.60 for CCC. Approximately 60% of the nutrients had a CCC above 0.60. If we estimated the validity by comparing R24 with FFQ.2, we observed a slight decrease in the correlation values obtained (0.67, 0.63 and 0.54), probably because the time elapsed between the two surveys exceeded 12 weeks [2, 26].

As for reproducibility, better results were obtained, with an average Pearson’s correlation coefficient of 0.67, 0.64 ICC and 0.58 CCC. It should be noted that, in this case, 70.6% of the nutrients obtained a CCC above 0.6.

Only vitamins A and D and calcium had a much lower correlation. This is probably due to the lack of nutritional information about these vitamins and this mineral in the South American food composition tables [33, 34] added to the Peruvian Amazon food database and used for this validation. The lack of information about common foods in food composition tables is considered a source of error in the validation of FFQs [2].

After adjusting the data by the residual method, a slight fall was produced in the ICC for almost all nutrients, as shown in other studies [10, 23, 35–37].

Our results are higher than those obtained in the studies performed by Dehgham [8, 38], Elorriaga [31], Satvinder [35] and Marcinkevage [39] and similar to others, such as the one carried out by Jackson [9] on the Jamaican population group, which obtained an ICC range between 0.5 and 0.88. Nor was it possible in this study to establish a correlation for fat-soluble vitamins. Another study performed on a New Zealand population group by Wong JE, obtained a Spearman’s correlation coefficient of 0.71 and an ICC of 0.69 when estimating reproducibility [40].

The Bland–Altman plot, based on a graphical interpretation, was used to obtain further information about the relationship between the FFQs and the results obtained via the R24. The results we observed are similar to the ones shown on the studies conducted by Trinidad [10], Zapata [32], Goni [41] and De Salvo [42], where a small number of individuals fell outside the recommended limits, confirming an acceptable level of agreement between both methods.

The classification capacity obtained by the FFQ was greater than the one observed in other studies [25, 31, 32, 43, 44] when comparing the extreme misclassification measured with FFQ.1 and the average of the three R24. However, it should be noted that some studies, for comparison purposes, grouped the participants in tertiles [25] or quartiles [45] instead of quintiles, as was the case in our study.

**Fig. 3:**
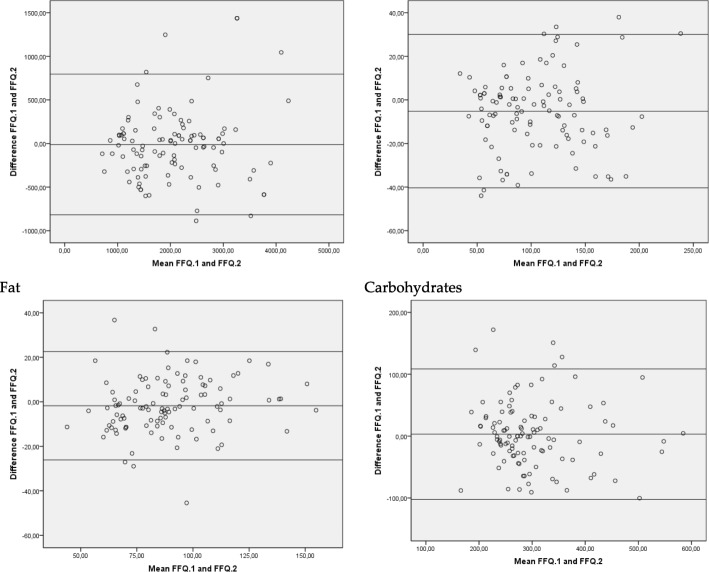
Bland–Altman plots. Reproducibility

We can, therefore, confirm that the correlation coefficients indicate a good relationship to establish both validity and reproducibility, since they are within the values considered acceptable (0.5–0.8) according to Cade et al. and Willet [2, 46] (Fig. 3).

## Conclusions

We conclude that the FFQ employed is a useful and valid tool to estimate the dietary intake of the Peruvian Amazon population, and thus, it can be used in future studies that link dietary patterns to chronic diseases.

## Limitations

The main limitation of this research is the fact that it was impossible to use accepted biomarkers as a reference value for the validation of the FFQ. However, numerous researchers also used R24, just like us [8, 9, 20, 23, 31, 32, 35, 38, 39, 44, 45].

Another limitation is the lack of information about certain nutrients in the Peruvian food composition tables [47] and in other databases around South America [33, 34]. Even though food composition tables were specifically written for this area, these nutrients could not be evaluated using the FFQ. To fill this information gap, the most representative foods need to be analysed in the laboratory [48].

## Supplementary information

**Additional file 1.** Annexed 1 food frequency questionnaire for Peruvian amazon population.

## Data Availability

The datasets used and/or analysed during the current study are available from the corresponding author on reasonable request.

## Abbreviations

CCC: Lin’s concordance correlation coefficient
FFQ: Food frequency questionnaire
ICC: Intraclass correlation coefficient
INEI (in Spanish): Peruvian National Institute of Statistics and Computing
R24: 24-h recall
