# Supplementary material for: Design and validation of a food frequency questionnaire (FFQ) for the nutritional evaluation of food intake in the Peruvian Amazon
Source: J Health Popul Nutr. 2019 Dec 19;38:47. doi: 10.1186/s41043-019-0199-8 (PMC6923861; doi:10.1186/s41043-019-0199-8)
Supplement: Supplementary file 1 — Additional file 1. Annexed 1 food frequency questionnaire for Peruvian amazon population. [file 41043_2019_199_MOESM1_ESM.docx]

**ANNEXED 1. FOOD FREQUENCY QUESTIONNAIRE FOR PERUVIAN AMAZON POPULATION**

**CUESTIONARIO DE FRECUENCIA DE CONSUMO DE ALIMENTOS PARA LA AMAZONÍA PERUANA**

*Por favor, marque una única opción para cada alimento.*

*Para cada alimento marque el recuadro que marca la frecuencia de consumo por término medio durante el año pasado.*

*Debe tenerse en cuenta las variaciones de alimento por temporadas, sobre todo para frutas y verduras, y hacerse una estimación del consumo. Por ejemplo, si se come sandía todos los días una vez durante el mes de septiembre el consumo estimado será de 1-3 veces al mes.*

1. **PRODUCTOS LÁCTEOS.**

| SEMANALMENTE | | | DIARIAMENTE | | | |  |  |  |
| --- | --- | --- | --- | --- | --- | --- | --- | --- | --- |
| **LÁCTEOS** | Nunca o casi nunca | 1-3 al mes | 1 | 2-4 | 5-6 | 1 | 2-3 | 4-6 | +6 |
| 1.Leche en polvo descremada (1 cucharada, 5-10g) |  |  |  |  |  |  |  |  |  |
| 2.Leche en polvo entera (1 cucharada, 5-10g) |  |  |  |  |  |  |  |  |  |
| 3.Leche evaporada entera (Media taza) |  |  |  |  |  |  |  |  |  |
| 4.Leche evaporada descremada (Media taza) |  |  |  |  |  |  |  |  |  |
| 5..Leche semidescremada UHT (1 taza, 200ml) |  |  |  |  |  |  |  |  |  |
| 6.Leche descremada UHT (1 taza, 200ml) |  |  |  |  |  |  |  |  |  |
| 7.Queso fresco (50g) |  |  |  |  |  |  |  |  |  |
| 8.Quesos grasos y semigrasos (50g) |  |  |  |  |  |  |  |  |  |
| 9.Yogurt natural (Unidad, 125g) |  |  |  |  |  |  |  |  |  |
| 10.Yogurt de frutas (Unidad, 125g) |  |  |  |  |  |  |  |  |  |
| 11.Mingados (1 taza, 200g) |  |  |  |  |  |  |  |  |  |
| 12.Helados (1 bola, 75g) |  |  |  |  |  |  |  |  |  |
| 13. Otros |  |  |  |  |  |  |  |  |  |

1. **HUEVOS, CARNES, PESCADOS Y MARISCOS.** Por ración o plato de 100-150g, mientras no se indique otra cantidad.

| SEMANALMENTE | | | DIARIAMENTE | | | |  |  |  |
| --- | --- | --- | --- | --- | --- | --- | --- | --- | --- |
| **HUEVOS, CARNES, PESCADOS Y MARISCOS** | Nunca o casi nunca | 1-3 AL MES | 1 | 2-4 | 5-6 | 1 | 2-3 | 4-6 | +6 |
| 1.Huevo de gallina (Unidad, 65g) |  |  |  |  |  |  |  |  |  |
| 2.Huevo de charapa (Unidad, 50g ) |  |  |  |  |  |  |  |  |  |
| 3.Pollo, pavo o gallina con piel (1 ración) |  |  |  |  |  |  |  |  |  |
| 4.Pollo, pavo o gallina sin piel (1 ración) |  |  |  |  |  |  |  |  |  |
| 5.Carne de res (1 ración) |  |  |  |  |  |  |  |  |  |
| 6.Corazón de res (1 ración) |  |  |  |  |  |  |  |  |  |
| 7.Carne de chancho (1 ración) |  |  |  |  |  |  |  |  |  |
| 8.Carne de lagarto (1 ración) |  |  |  |  |  |  |  |  |  |
| 9.Carnes procesadas: chorizo, salchichas, etc. (50g) |  |  |  |  |  |  |  |  |  |
| 10.Tocino (50g) |  |  |  |  |  |  |  |  |  |
| 11.Hígado de res (1 ración) |  |  |  |  |  |  |  |  |  |
| 12.Menudencias (1 ración) |  |  |  |  |  |  |  |  |  |
| 13.Hamburguesa (1, 50g) |  |  |  |  |  |  |  |  |  |
| 14.Pescado magro: corvina, doncella, bagre, et. (1 ración) |  |  |  |  |  |  |  |  |  |
| 15.Pescado graso: palometa, sábalo, carachama, (1 ración) |  |  |  |  |  |  |  |  |  |
| 16. Pescado salado(1 ración) |  |  |  |  |  |  |  |  |  |
| 17. Mariscos: camarón, mejillón, calamar, pulpo (1 ración, 200g) |  |  |  |  |  |  |  |  |  |
| 18. Otros |  |  |  |  |  |  |  |  |  |

1. **VERDURAS Y HORTALIZAS.** Un plato o ración de 200g mientras no se indique otra cantidad.

| SEMANALMENTE | | | DIARIAMENTE | | | |  |  |  |
| --- | --- | --- | --- | --- | --- | --- | --- | --- | --- |
| **VERDURAS, HORTALIZAS, TUBÉRCULOS Y PLÁTANO VERDE** | Nunca o casi nunca | 1-3 AL MES | 1 | 2-4 | 5-6 | 1 | 2-3 | 4-6 | +6 |
| 1.Espinaca, acelgas (1 ración) |  |  |  |  |  |  |  |  |  |
| 2.Lechuga (100g) |  |  |  |  |  |  |  |  |  |
| 3.Chonta (50g) |  |  |  |  |  |  |  |  |  |
| 4.Tomate (1, 150g) |  |  |  |  |  |  |  |  |  |
| 5.Pepino (1 ración) |  |  |  |  |  |  |  |  |  |
| 6.Brócoli, col, coliflor, repollo( ración) |  |  |  |  |  |  |  |  |  |
| 7. Zanahoria, calabaza (1 ración) |  |  |  |  |  |  |  |  |  |
| 8.Beterraga (1, 140g) |  |  |  |  |  |  |  |  |  |
| 9.Ají dulce (150g) |  |  |  |  |  |  |  |  |  |
| 10.Ají rocoto (100g) |  |  |  |  |  |  |  |  |  |
| 11.Ají charapita (20g) |  |  |  |  |  |  |  |  |  |
| 12.Hongos (100g) |  |  |  |  |  |  |  |  |  |
| 13.Palta 1 (150g) |  |  |  |  |  |  |  |  |  |
| 14.Cebolla. Media (50g) |  |  |  |  |  |  |  |  |  |
| 15.Culantro (una pizca) |  |  |  |  |  |  |  |  |  |
| 16.Vainas (50g) |  |  |  |  |  |  |  |  |  |
| 17. Papa (1 ración) |  |  |  |  |  |  |  |  |  |
| 18. Camote (1 ración) |  |  |  |  |  |  |  |  |  |
| 19. Yuca (1 ración) |  |  |  |  |  |  |  |  |  |
| 20. Sachapapa (1 ración) |  |  |  |  |  |  |  |  |  |
| 21. Pijuallo (1 ración) |  |  |  |  |  |  |  |  |  |
| 22. Plátano verde (1 ración) |  |  |  |  |  |  |  |  |  |
| 23. Otros |  |  |  |  |  |  |  |  |  |

1. **FRUTAS**. Una pieza o ración de 100-150g

| SEMANALMENTE | | | DIARIAMENTE | | | |  |  |  |
| --- | --- | --- | --- | --- | --- | --- | --- | --- | --- |
| **FRUTAS** | Nunca o casi nunca | 1-3 AL MES | 1 | 2-4 | 5-6 | 1 | 2-3 | 4-6 | +6 |
| 1.Naranja (1), limón (2), mandarina (2) |  |  |  |  |  |  |  |  |  |
| 2.Sandía (1 porción) |  |  |  |  |  |  |  |  |  |
| 3.Aguaje (3, 130g) |  |  |  |  |  |  |  |  |  |
| 4.Toronja (1) |  |  |  |  |  |  |  |  |  |
| 5.Plátano maduro (1) |  |  |  |  |  |  |  |  |  |
| 6. Manzana, pera (1, 200g) |  |  |  |  |  |  |  |  |  |
| 7.Mango (1) |  |  |  |  |  |  |  |  |  |
| 8.Piña(1 ración) |  |  |  |  |  |  |  |  |  |
| 9.Papaya (1 ración) |  |  |  |  |  |  |  |  |  |
| 10.Melón (1 ración) |  |  |  |  |  |  |  |  |  |
| 11. Anona (1 ración) |  |  |  |  |  |  |  |  |  |
| 12. Caimito (1 ración) |  |  |  |  |  |  |  |  |  |
| 13.Coco (1 ración) |  |  |  |  |  |  |  |  |  |
| 14.Fresa (100g) |  |  |  |  |  |  |  |  |  |
| 15.Granadilla (3, 150g) |  |  |  |  |  |  |  |  |  |
| 16.Cocona (1, 60g) |  |  |  |  |  |  |  |  |  |
| 17.Charichuelo (100g) |  |  |  |  |  |  |  |  |  |
| 18. Uva (100g) |  |  |  |  |  |  |  |  |  |
| 19.Carambola, camucamu, maracuyá, cocona, copoazú (vaso de refresco) |  |  |  |  |  |  |  |  |  |
| 20.Otras frutas |  |  |  |  |  |  |  |  |  |

**5. CEREALES, MENESTRAS Y FRUTOS SECOS.** Por ración de 150g mientras no se indique otra cantidad.

| SEMANALMENTE | | | DIARIAMENTE | | | |  |  |  |
| --- | --- | --- | --- | --- | --- | --- | --- | --- | --- |
| **CEREALES Y MENESTRAS** | Nunca o casi nunca | 1-3 AL MES | 1 | 2-4 | 5-6 | 1 | 2-3 | 4-6 | +6 |
| 1.Arroz blanco (60g en crudo) |  |  |  |  |  |  |  |  |  |
| 2.Avena (1 taza) |  |  |  |  |  |  |  |  |  |
| 3.Cañihua, kiwicha, quinua (1 taza) |  |  |  |  |  |  |  |  |  |
| 4.Cebada (1 taza) |  |  |  |  |  |  |  |  |  |
| 5.Copos de trigo (1 taza) |  |  |  |  |  |  |  |  |  |
| 6.Cereales azucarados (1 taza) |  |  |  |  |  |  |  |  |  |
| 7.Pan blanco, pan de molde, pan de leche (20g, 1 rebanada) |  |  |  |  |  |  |  |  |  |
| 8.Pan integral (20g, 1 rebanada) |  |  |  |  |  |  |  |  |  |
| 9.Pasta: fideos, macarrones, tallarín, etc. (60g en crudo) |  |  |  |  |  |  |  |  |  |
| 10.Harina de trigo, harina de maíz, fariña. (100g) |  |  |  |  |  |  |  |  |  |
| 11. Alverjas (1 ración) |  |  |  |  |  |  |  |  |  |
| 12.Lentejas (1 ración) |  |  |  |  |  |  |  |  |  |
| 13. Frijol (1 ración) |  |  |  |  |  |  |  |  |  |
| 14. Canchitas (50g) |  |  |  |  |  |  |  |  |  |
| 15. Maní tostado (50g) |  |  |  |  |  |  |  |  |  |
| 16.Pasas, dátiles, orejones (50g) |  |  |  |  |  |  |  |  |  |

1. **GRASAS Y ACEITES.** 1 cucharada sopera (10ml)

| SEMANALMENTE | | | DIARIAMENTE | | | |  |  |  |
| --- | --- | --- | --- | --- | --- | --- | --- | --- | --- |
| **GRASAS Y ACEITES** | Nunca o casi nunca | 1-3 AL MES | 1 | 2-4 | 5-6 | 1 | 2-3 | 4-6 | +6 |
| 1.Aceite vegetal. Mezcla soya, algodón, girasol…(1 ración) |  |  |  |  |  |  |  |  |  |
| 2.Aceite de soya (1 ración) |  |  |  |  |  |  |  |  |  |
| 3.Aceite de girasol (1 ración) |  |  |  |  |  |  |  |  |  |
| 4.Aceite de Sacha Hinchi (1 ración) |  |  |  |  |  |  |  |  |  |
| 5.Aceite de palma (1 ración) |  |  |  |  |  |  |  |  |  |
| 6.Aceite de oliva (1 ración) |  |  |  |  |  |  |  |  |  |
| 7.Mantequilla con sal (1 porción individual, 12g) |  |  |  |  |  |  |  |  |  |
| 8.Margarina (1 porción individual, 12g) |  |  |  |  |  |  |  |  |  |
| 9.Manteca de cerdo (10g) |  |  |  |  |  |  |  |  |  |

1. **CREMAS**. 1 cucharada, 10-15g.

| SEMANALMENTE | | | DIARIAMENTE | | | |  |  |  |
| --- | --- | --- | --- | --- | --- | --- | --- | --- | --- |
| **CREMAS** | Nunca o casi nunca | 1-3 AL MES | 1 | 2-4 | 5-6 | 1 | 2-3 | 4-6 | +6 |
| 1.Salsa de ají (de cebolla, de cocona, etc.) |  |  |  |  |  |  |  |  |  |
| 2.Mayonesa |  |  |  |  |  |  |  |  |  |
| 3.Ketchup |  |  |  |  |  |  |  |  |  |
| 4.Mostaza |  |  |  |  |  |  |  |  |  |

1. **DULCES Y SNACKS.** Por ración indicada en la tabla.

| SEMANALMENTE | | | DIARIAMENTE | | | |  |  |  |
| --- | --- | --- | --- | --- | --- | --- | --- | --- | --- |
| **DULCES Y SNAKS** | Nunca o casi nunca | 1-3 AL MES | 1 | 2-4 | 5-6 | 1 | 2-3 | 4-6 | +6 |
| 1.Azúcar (1 cucharadita) |  |  |  |  |  |  |  |  |  |
| 2.Miel (1 cucharada) |  |  |  |  |  |  |  |  |  |
| 3.Mermelada (1 cucharada) |  |  |  |  |  |  |  |  |  |
| 4.Galletas saladas (4 unidades, 50g) |  |  |  |  |  |  |  |  |  |
| 5.Galletas rellenas de chocolate o vainilla (2 unidades, 50g) |  |  |  |  |  |  |  |  |  |
| 6.Tortas o queques (1 porción) |  |  |  |  |  |  |  |  |  |
| 7.Chocolates (50g) |  |  |  |  |  |  |  |  |  |
| 8.Caramelos, bombones, paletas (unidad) |  |  |  |  |  |  |  |  |  |
| 9.Curichis y chupetes (unidad) |  |  |  |  |  |  |  |  |  |
| 10.Frutos secos fritos (1 bolsa, 50g) |  |  |  |  |  |  |  |  |  |
| 11.Patatas chips, chifles, popcorn (1 bolsa, 50g) |  |  |  |  |  |  |  |  |  |
| 12. Otros dulces y/o snaks. |  |  |  |  |  |  |  |  |  |

8. **BEBIDAS.** Por un vaso de 250ml a no ser que haya otra opción.

| SEMANALMENTE | | | DIARIAMENTE | | | |  |  |  |
| --- | --- | --- | --- | --- | --- | --- | --- | --- | --- |
| **BEBIDAS** | Nunca o casi nunca | 1-3 AL MES | 1 | 2-4 | 5-6 | 1 | 2-3 | 4-6 | +6 |
| 1.Agua del grifo (1 vaso) |  |  |  |  |  |  |  |  |  |
| 2.Agua mineral (1 vaso) |  |  |  |  |  |  |  |  |  |
| 3.Gaseosas (1 vaso) |  |  |  |  |  |  |  |  |  |
| 4. Gaseosas light o cero (1 vaso) |  |  |  |  |  |  |  |  |  |
| 5.Refrescos (1 vaso) |  |  |  |  |  |  |  |  |  |
| 6. Jugos embotellados y frugos (1 vaso) |  |  |  |  |  |  |  |  |  |
| 7.Cerveza (1 vaso) |  |  |  |  |  |  |  |  |  |
| 8.Jugos (1 vaso) |  |  |  |  |  |  |  |  |  |
| 9.Destilados y tragos: ron, pisco, vodka, whisky (50 cc) |  |  |  |  |  |  |  |  |  |
| 10.Licores (50cc) |  |  |  |  |  |  |  |  |  |
| 11.Café (taza, 200ml) |  |  |  |  |  |  |  |  |  |
| 12.Té o infusiones (1 taza, 200ml) |  |  |  |  |  |  |  |  |  |
| 13. Otra bebida |  |  |  |  |  |  |  |  |  |

1. **CONDIMENTOS.** Una cucharada de café.

| SEMANALMENTE | | | DIARIAMENTE | | | |  |  |  |
| --- | --- | --- | --- | --- | --- | --- | --- | --- | --- |
| **CONDIMENTOS** | Nunca o casi nunca | 1-3 AL MES | 1 | 2-4 | 5-6 | 1 | 2-3 | 4-6 | +6 |
| 1.Sal |  |  |  |  |  |  |  |  |  |
| 2.Ajino |  |  |  |  |  |  |  |  |  |
| 3.Palillo |  |  |  |  |  |  |  |  |  |
| 4. Cillao |  |  |  |  |  |  |  |  |  |
| 5.Tabletas de caldo |  |  |  |  |  |  |  |  |  |
| 6. Pimienta |  |  |  |  |  |  |  |  |  |
| 7.Vinagre |  |  |  |  |  |  |  |  |  |
| 8.Ajo |  |  |  |  |  |  |  |  |  |
| 9.Comino |  |  |  |  |  |  |  |  |  |
| 10.Canela |  |  |  |  |  |  |  |  |  |
| 11.Otros |  |  |  |  |  |  |  |  |  |
